# Splice-Switching Antisense Oligonucleotides as a Targeted Intrinsic Engineering Tool for Generating Armored Redirected T Cells

**DOI:** 10.1089/nat.2020.0905

**Published:** 2021-03-25

**Authors:** Erica Ceccarello, Tommaso Tabaglio, Sarene Koh, Vincent Oei, Winnie Teo, Owen Julianto Jonathan, Andrea Pavesi, Qingfeng Chen, Antonio Bertoletti, Keng Boon Wee, Ernesto Guccione

**Affiliations:** ^1^ Institute of Molecular and Cell Biology, Agency for Science, Technology and Research (A*STAR), Singapore, Singapore.; ^2^ Duke-NUS Medical School, Singapore, Singapore.; ^3^ IMMUNOA Pte Ltd, Singapore, Singapore.; ^4^ Lion TCR Pte Ltd, Singapore, Singapore.; ^5^ Singapore Immunology Network, Agency for Science and Technology (A*STAR), Singapore, Singapore.; ^6^ Department of Oncological Sciences and Pharmacological Sciences, Center for Therapeutics Discovery, Tisch Cancer Institute, Icahn School of Medicine at Mount Sinai, New York, New York, USA.

**Keywords:** splice switching antisense oligonucleotides (SSOs), HBV^+^HCC, TCR-redirected T cells

## Abstract

Modification of specificity of T cells for the use in adoptive transfer (CAR- or TCR-redirected T cells) has revolutionized the therapy of liquid tumors and some infectious diseases. However, several obstacles are still hampering the efficacy of such potent therapy, hence concurrent modification of the function is also required to obtain successful results. Here we show the use of splice-switching antisense oligonucleotides (SSOs) as a tool to transiently modify T cell function. We demonstrate the possibility to transfect SSOs and an exogenous TCR into primary human T cells in the same electroporation reaction, without affecting viability and function of the transfected T lymphocytes. Moreover, we show that SSOs targeting T cell-specific mRNAs induce the skipping of the targeted exons, and the reduction of the protein and consequent modification of T cell function. This technical work paves the way to the use of SSOs in immune cells, not only for the knockdown of the functional isoform of the targeted proteins, but also for the protein manipulation by elimination of specific domains encoded by targeted exons.

## Introduction

Adoptive T cell transfer is a versatile cell therapy modality that has the potential to address critical medical needs from chronic infections to oncology. Through the engineering of effector T cells with specific receptors, in the form of chimeric antigen receptors (CARs) or classical T cell receptors (TCRs), one can direct the lytic action of CD8^+^ T cells against a specific target. As examples, viral-infected host cells presenting viral antigens have been targeted by CAR/TCR-redirected T cells against human immunodeficiency virus, hepatitis B virus (HBV), hepatitis C virus, human cytomegalovirus, or opportunistic fungal infections [1–8]. Moreover, cancer cells have been targeted using CAR/TCR specific for antigens expressed on the cell of origin (eg, CD19 for B cell leukemia and lymphoma) [9,10] or targeting specific tumor antigens of self or viral origins [11–17].

To unleash the full clinical potential of adoptive T cell therapy beyond liquid tumors, two levels of cell engineering can be exploited—extrinsic and intrinsic. Extrinsic engineering, in the form of synthetic receptors, has been the hallmark of the first-generation CAR/TCR-redirected T cells. The second generation is differentiated by the modification and/or modulation of T cell endogenous factors, to induce functional features of effector T cells that are most propitious for each clinical application. As an example, for antiviral therapy the cytolytic activity and inflammatory induction of redirected T cells need to be cautiously calibrated to avert massive lysis of the infected targeted organs, and/or cytokine release syndrome [18]. On the contrary, in the context of solid tumors, first-generation adoptive CAR/TCR-redirected T cells are not equipped to bypass inhibitory factors in the tumor microenvironment or to specifically reach the target in specific organs. For this reason, efforts are ongoing to engineer TCR/CAR-redirected T cells with improved trafficking, secreting immune checkpoint inhibitors or stimulatory cytokines [19–23].

The molecular tools used in the intrinsic engineering of T cells for adoptive transfer include gene knock-in with a lenti/retroviral vector, gene knockout using TALEN or CRISPR/Cas9 technologies, and suppression of gene expression with siRNAs or GAPmers [24–27].

Here we propose and demonstrate the feasibility and flexibility of splice-switching antisense oligonucleotides (SSOs) for the intrinsic modification of T cell functions and to generate what we define armored redirected T cells (ART cells). An SSO modulates target transcript splicing by denying access of specific RNA binding proteins (RNA-BPs) to their splice-regulatory motifs through steric hindrance. Synthesized as a short single-stranded ribonucleic acid whose bases and backbones are chemically modified, the SSO is directed to bind complementarily to a pre-mRNA target sequence containing splicing silencers or enhancers motifs [28,29]. We and others have demonstrated the flexibility of SSO application in the suppression of transcript abundance by inducing nonsense-mediated decay [30], correction of aberrant and mis-splicing events [31–37], and selection of alternate splicing [38–41].

Specifically, we designed and validated novel SSOs modulating the splicing of three T cell-specific genes, namely interferon-γ (IFN-γ), perforin (PRF), and granzyme B (GZMB), for the targeted intrinsic engineering of cytotoxicity and cytokine production of primary human T cells. We demonstrate that SSOs can be efficiently transfected into primary human T cells concurrently with a synthetic TCR mRNA to create ART cells. This work paves the way for the development of a wider array of SSOs modulating T cell-relevant genes in different ways, which are key to improve adoptive T cell immunotherapy.

## Materials and Methods

### Institutional review board

Approval: IRB No. H-17-023E issued by National University of Singapore (NUS). All patients gave written informed consent.

### Ficoll-Paque blood separation

Peripheral blood mononuclear cells (PBMCs) from healthy donors were obtained from full blood using Ficoll-Paque (GE Health Care, Chicago, IL) centrifugation. Ficoll-Paque (10 mL) was placed at the bottom of a 50 mL Falcon tube and blood was slowly layered above. After being centrifuged (600 *g* for 30 min at 18°C, no brakes), a layer of PBMCs would be visible and collected for further experiments.

### T cell activation and expansion from PBMCs

Frozen PBMCs were thawed adding 14 mL of warm Hanks' balanced salt solution (HBSS; ThermoFisher Scientific, Waltham, MA) in a dropwise manner. After wash (427 RCF, 5 min, room temperature [RT]), PBMCs were cultured in AIM-V medium (ThermoFisher Scientific) supplemented with human AB serum (Sigma-Aldrich, St. Louis, MO) at a concentration of 1.5–2 × 10^6^ cells/mL. T cells were activated adding 50 ng/mL of anti-CD3 (eBioscience, San Diego, CA) and 600 IU/mL of recombinant human interleukin-2 (rhIL-2; Miltenyi Biotec, Bergisch Gladbach, Germany).

Resting T cells were separated from PBMCs using the pan T cell isolation kit (Miltenyi Biotec) 1 day after thawing. Resting T cells were cultured in AIM-V supplemented with 2% AB serum and 100 U/mL rhIL-2.

### HBV-specific TCR mRNA production

We derived the TCR construct from a pUC57-s183cys b2Aa vector that we had previously made, and subcloned it into the pVAX1 vector [42]. The plasmid was propagated and purified from *Escherichia coli* using the One Shot Top10 *E. coli* kit (ThermoFisher Scientific), purified using QIAGEN EndoFree Plasmid Maxi Kit (Qiagen, Hilden, Germany), and linearized using the *Xba*I restriction enzyme (New England Biolabs, Ipswich, MA). The linearized DNA was used to produce the TCR mRNA using the mMESSAGE mMACHINE T7 Ultra kit (ThermoFisher Scientific) following the manufacturer's instructions.

### Splicing-modifying antisense oligonucleotides

SSOs were synthesized by Integrated DNA Technologies (Coralville, IA) with a phosphorothioate backbone and 2′-*O*-methyl ribose modifications in each position. The SSOs were resuspended in water at a final concentration of 1 mM or 500 μM and kept frozen at −20°C. The SSOs were added in the electroporation mix together with the T cells (and the TCR mRNA, eventually) in electroporation buffer at the desired concentration.

Detailed information about the utilized SSOs are given in “Modifying T lymphocytes function with Antisense Oligonucleotides (ASOs) for personalized immune therapy” (PCT/SG2018/050313; 10201705285S (IMC/Z/09724); filing date June 27, 2017; priority date June 27, 2016; licensed to IMMUNOA Pte Ltd on September 14, 2018).

### Electroporation

T cells were transfected using electroporation method using the 4DNucleofector™ System (Lonza, Basel, Switzerland). T cells were washed twice with phosphate-buffered saline (PBS) and electroporated using the P3 Primary Cell 4D-Nucleofector^®^ X kit following the manufacturer's instructions with a customized electroporation program. Electroporated T cells were then resuspended in warm AIM-V medium supplemented with 10% AB serum and 100 U/mL rhIL-2.

Usually, 5–10 × 10^6^ cells were electroporated in each reaction. The TCR mRNA was added at 2 μg/10^6^ T cells; SSOs were added at different concentrations ranging from 0.15 to 0.5 femtomoles/T cell.

### Cell line culture

HepG2.2.15 were cultured in Dulbecco's modified Eagle's medium (DMEM; ThermoFisher Scientific) supplemented with 10% v/v heat inactivated fetal bovine serum (FBS; ThermoFisher Scientific), 2% v/v penicillin/streptomycin, 1% v/v MeM nonessential amino acids (ThermoFisher Scientific), 1 mM sodium pyruvate (ThermoFisher Scientific), and 200 μg/mL Geneticin reagent (ThermoFisher Scientific) to select for transgene expressing cells. THP-1 cells are cultured in Roswell Park Memorial Institute Medium (RPMI; ThermoFisher Scientific) supplemented with 10% v/v heat inactivated FBS and 1% v/v penicillin/streptomycin.

### Real-time cytotoxicity assay

The cytotoxicity assays were performed using the xCELLigence^®^ RTCA DP (ACEA Biosciences, Inc., San Diego, CA) following the manufacturer's specifications. In brief, 10^5^ HepG2.2.15 cells/180 μL were seeded in the specific plate (in their growth medium) and were let to adhere for 24 h. At the time of effector addition, 150 μL of medium were removed and replaced with T cells resuspended in AIM-V 2% AB serum, at a fixed effector:target (E:T) ratio. The acquisition was started at the seeding of the targets and continued for 48 h after T cell addition; the impedance measurements were acquired every 15 min. The Cell Index is a measurement of the impedance measured in each well, and the Normalized Cell Index was obtained by normalizing the Cell Index of each well to the Cell Index at a specific time point (the sweep before T cell addition); the area under the curve (AUC) was obtained using the GraphPad 7 algorithm (San Diego, CA).

### Coculture experiments (PD-L1 detection)

Experiments of coculture were performed using TCR-redirected T cells specific for S183–191 peptide of HBV envelope. THP-1 cells were resuspended at 2 M/mL in medium and s183–191 peptides were added at a concentration of 1 μg/mL, at room temperature; after 1 h, the supernatant was removed, and the cells were carefully rinsed twice with warm HBSS. HBV-specific TCR-redirected T cells were cocultured with the targets in AIM-V 2% AB serum for 5 h at different E:T ratios. The supernatants of the coculture were then collected and transferred onto other THP-1 cells for 8 h. The THP-1 cells were then collected and stained for the presence of PD-L1.

### Surface and intracellular staining

After culture, cells were collected and washed once (427 RCF, 3 min, 4°C) with PBS in 96-well plated (V-bottomed). Live/dead staining was performed in PBS for 10 min at RT, followed by two washes with cold PBS; MHC-I dextramer staining was performed in staining buffer (SB; PBS supplemented with 1% bovine serum albumin [BSA]; Sigma-Aldrich) and 0.1% sodium azide (Sigma-Aldrich) at RT for 15 min followed by one wash in SB; surface staining was carried out in SB for 30 min on ice, followed by two washes and 20 min of fixing and permeabilization using Cytofix/Cytoperm solution (BD, Franklin Lakes, NJ) on ice; finally, intracellular staining was performed in Permwash buffer (PBS supplemented with 1% BSA, 0.1% sodium azide, and 0.1% saponin; Sigma-Aldrich) for 30 min on ice. After intracellular staining, the sample was washed twice in Permwash buffer, and resuspended in PBS supplemented with 1% formaldehyde for flow cytometry acquisition. The samples were acquired on LSRII (BD), and analyzed using Kaluza (London, United Kingdom) or FlowJo (Ashland, OR) software.

### RNA extraction and polymerase chain reaction

RNA extraction was performed using RNeasy Plus Micro kit (Qiagen) following the manufacturer's instructions. After extraction, the RNA was quantified using NanoDrop and was retro-transcribed using the cDNA iScript synthesis kit (BioRad, Hercules, CA). Polymerase chain reaction (PCR) experiments were performed to assess the effect of SSOs in skipping the target exon. Primers flanking the target exon were designed to obtain products of different sizes upon treatment with the specific SSO.

After PCR amplification, the products were run on an agarose gel (2%) and stained with SYBR Safe DNA Gel Stain (ThermoFisher Scientific) according to the manufacturer instructions. The gel was then imaged, and the images were analyzed using ImageJ software (NIH). The Percentage Spliced In was calculated after having analyzed the luminosity of the bands in the gel.

### Antibody list

CD3 (Biolegend, San Diego, CA), CD274 (BD), CD8 (BD), GZMB (BD), PRF (Diaclone, Besançon, France), tumor necrosis factor-α (BD), IFN-γ (ThermoFisher Scientific), s183–191 MHC-I dextramer (Immudex, Copenhagen, Denmark), and live/dead fixable stain kit (ThermoFisher Scientific).

## Results

### TCR mRNA and SSOs can be concurrently electroporated into T cells with no adverse effect

**FIG. 1.**
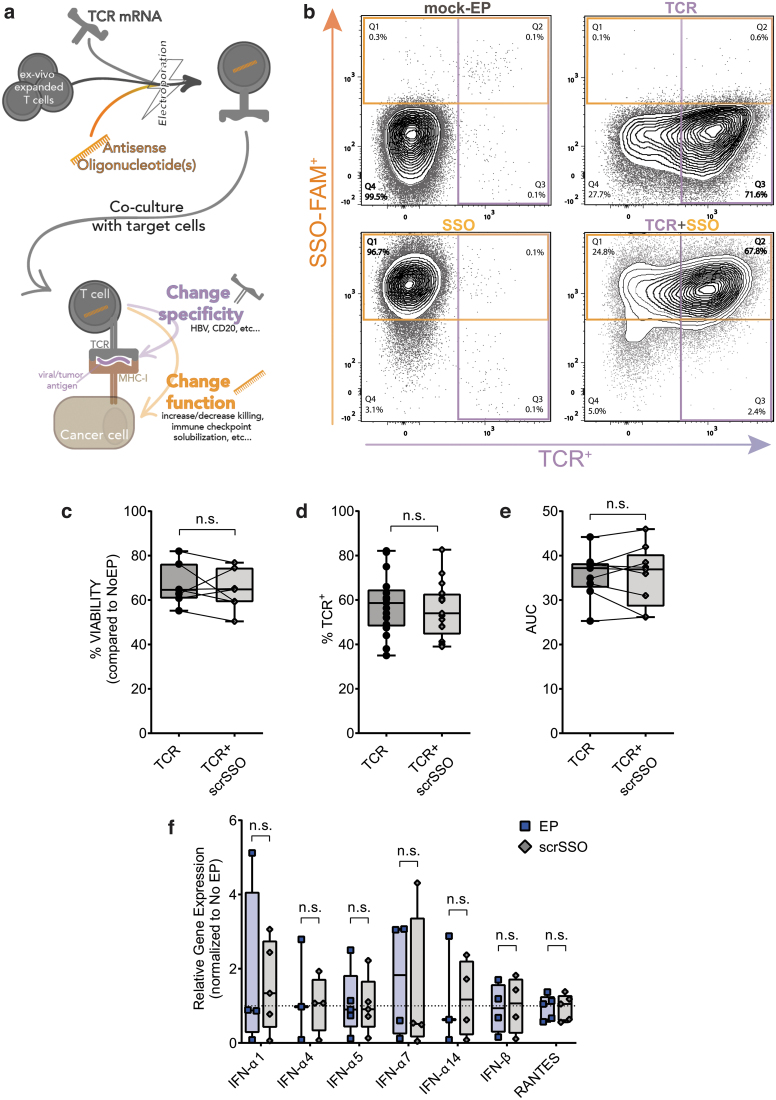
**(a)** Schematic idea of generation of ART cells. **(b)** Representative staining of T cells electroporated (*top left*), electroporated with HBV-specific TCR (*top right*), electroporated with a FAM-tagged scrambled SSO (*bottom left*) and with both transfectants (ART cells, *bottom right*). **(c)** Percentage viability of T cells mock electroporated or electroporated with scrSSO. **(d)** HBV-TCR expression in T cells electroporated with TCR mRNA or TCR mRNA and scrSSO. **(e)** Killing ability (xCELLigence RTCA) of T cells electroporated with TCR mRNA alone or TCR mRNA and scrSSO. **(f)** Expression of TLR-induced genes upon mock electroporation or electroporation of scrSSO 24 h after transfection (normalized to the expression of nonelectroporated T cells). ART cells, armored redirected T cells; TCR, T cell receptor; HBV, hepatitis B virus; n.s., not significant; SSO, splice-switching antisense oligonucleotide. Color images are available online.

To test our approach of concurrent extrinsic and intrinsic engineering to modulate specificity and function of primary human T cells, respectively, we electroporated in the same reaction an mRNA coding for a cognate-specific TCR and SSO (Fig. 1a).

T cell specificity can be transiently modified by electroporating an mRNA encoding for an exogenous TCR. We utilized an mRNA coding for a TCR specific for HBV epitopes restricted by HLA-class I molecule A0201; we had previously demonstrated that these mRNA-electroporated T cells transiently expressed HBV-TCR up to 72 h. The HBV-TCR expressing T cells were observed to lyse HCC cells expressing HBV antigens or inhibit HBV replication both *in vitro* and *in vivo* [4,42,43].

To assess the efficiency of SSO delivery in primary human T cells, an FAM-tagged SSO with a scrambled nontargeting sequence (scrSSO) was co-electroporated with the HBV-TCR mRNA [11]. An average of 70% cotransfection efficiency was achieved (Fig. 1b).

Of note, the concurrent transfection of HBV-TCR mRNA (TCR for short) and scrSSO does not impinge on the expected biophysical and biological properties of each other. The kinetics of HBV-TCR transfection (Supplementary Fig. S1a) and scrSSO transfection (Supplementary S1b) were not affected. Of importance, viability (Fig. 1c), TCR protein expression as assessed by MHC multimer staining in flow cytometry (Fig. 1d), and T cell antiviral activity as measured in a 2D killing assay (Fig. 1e) were not affected. The concomitant mRNA TCR and scrSSO electroporation was efficient not just on activated proliferating T cells but also in resting human primary T cells (Supplementary Fig. S1e, f). Moreover, scrSSO does not induce an elevation of TLR-related proinflammatory genes usually upregulated in the presence of naked nucleic acids (Fig. 1f) [44] up to 72 h after electroporation (Supplementary Fig. S1c, d).

### Generation of ART cells with reduced IFN-γ secretion capacity

IFN-γ is a proinflammatory cytokine secreted mainly by Th1-type T cells. Besides playing roles in antiviral function [45], IFN-γ can promote activation-induced cell death of T cells [46] and is the main inducer of both PD-L1 and PD-L2 expression [47], and thus participates in inducing an immunosuppressive environment [48,49].

**FIG. 2.**
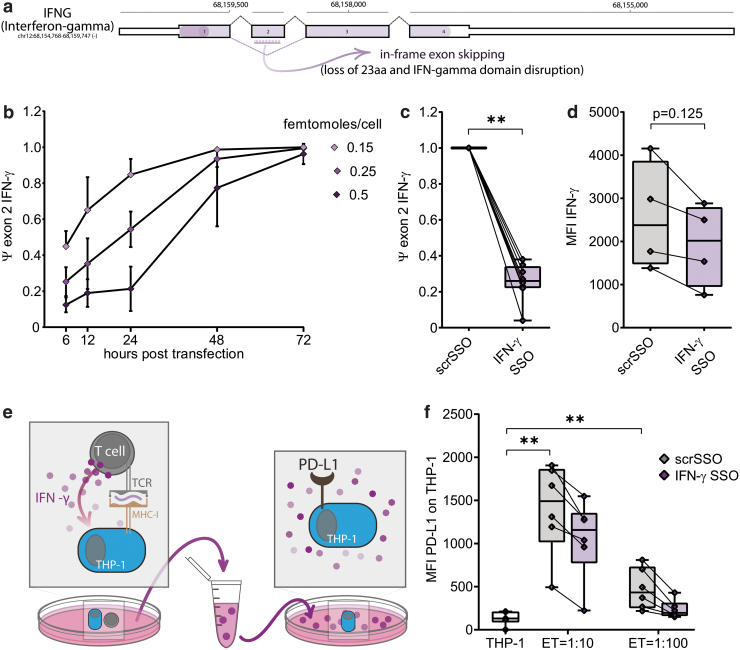
**(a)** Schematic of *IFNG* gene. **(b)** Exon skipping measured at 6–12–18–24–48–72 h after transfection with IFN-γ SSO (0.15–0.25–0.5 femtomoles/cell). **(c)** PSI (Percentage Spliced In) of exon 2 of IFN-γ mRNA 24 h after electroporation of scrSSO or IFN-γ SSO. **(d)** Mean fluorescence intensity of IFN-γ in T cells electroporated with scrSSO or IFN-γ SSO. **(e)** Schematic of the experiment: HBV-specific ART cells (transfected with scrSSO or IFN-γ SSO) were incubated with THP-1 cells presenting the specific HBV peptide (E:T ratio = 1:10 or 1:100). After incubation, the supernatants were collected and placed onto new THP-1 cells. After 8 h of culture, the expression of PD-L1 (induced by IFN-γ) was measured **(f)** PD-L1 expression on THP-1 cells cultured in supernatants deriving from **(e)**. **p < 0.01. E:T, effector:target; IFN- γ, interferon-γ. Color images are available online.

Exon 2 of *IFNG* codes for part of the “interferon-γ domain” (the specific cytokine domain), and we hypothesized that its exclusion will result in the expression of a shortened IFN-γ protein with attenuated cytokine function. We designed and synthesized an SSO to induce specific IFN-γ exon 2 skipping (Fig. 2a) and transfected through electroporation on activated primary human T cells, at three different concentrations. Figure 2b shows the temporal exon skipping efficiencies after transfection: exon 2 was skipped as early as 6 h after electroporation, and the level of exon skipping, as well as the duration of the effect, are dose dependent (as the skipping is reduced faster with lower doses of SSOs) (Supplementary Fig. S2c). At all the tested IFN-γ SSO concentrations, cell viability and TCR expression in primary human activated T cells were not affected (Supplementary S2a, b) while inducing skipping of exon 2 (Fig. 2c) 24 h after transfection (0.25 femtomoles/T cell). Similarly, viability and TCR expression are not affected by the SSO transfection in resting TCR-redirected T cells (Supplementary Fig. S2d, e) in the presence of *IFNG* exon 2 exclusion (Supplementary Fig. S2f).

Next, we tested using flow cytometry whether the alteration induced by IFN-γ SSO would lead to a reduction of IFN-γ 24 h after transfection with 0.25 femtomoles/cell (Fig. 2d). We then assessed the ability of the T cells supernatant to induce PD-L1 expression in a monocytic cell line (THP-1) [50]. IFN-γ ART cells resulted in monocytes producing up to 40% less PD-L1 protein when cultured with supernatants derived from activated IFN-γ ART cells (Fig. 2e, f), demonstrating a functional difference compared with the scrSSO controls.

### Generation of ART cells with reduced cytotoxic activity: PRF and GZMB

**FIG. 3.**
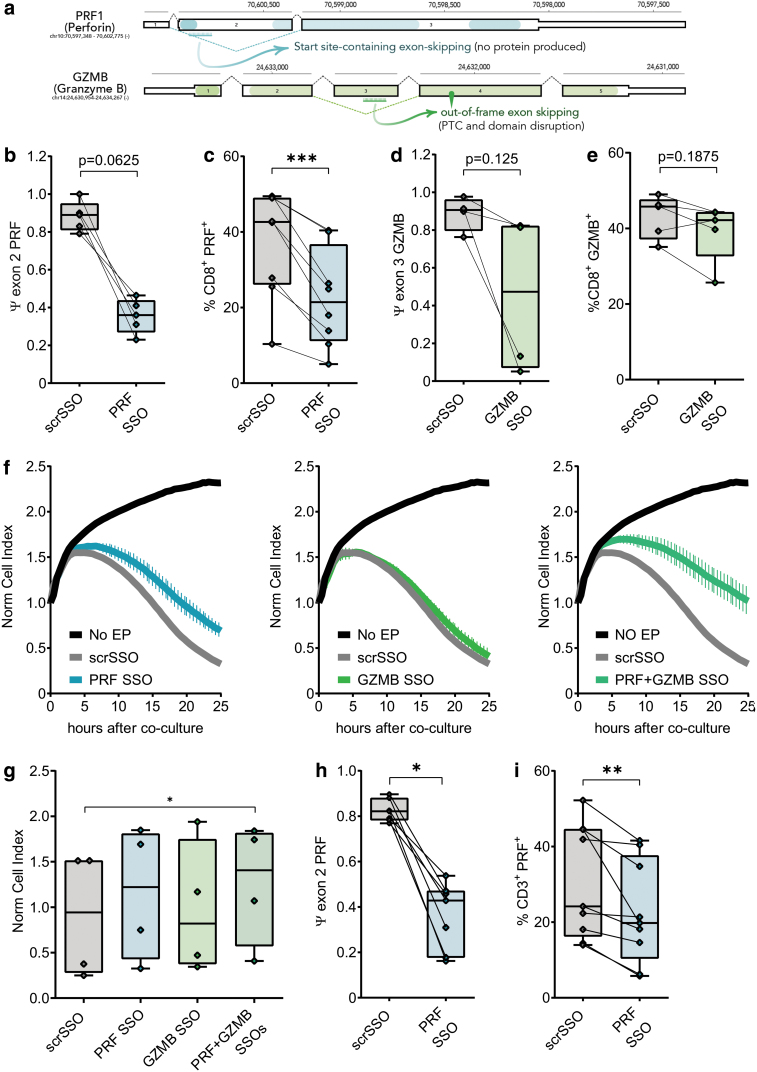
**(a)** Schematic of *PRF1* (perforin) and *GZMB* genes. **(b)** PSI of exon 2 from PRF mRNA in ART cells electroporated with either scrSSO or PRF SSO. **(c)** Flow cytometry staining of PRF in PRF ART cells and control. **(d)** PSI of exon 3 of GZMB upon electroporation of GZMB SSO. **(e)** Flow cytometry staining of GZMB in GZMB ART cells and control. **(f)** Representative curves of killing obtained in the xCELLigence^®^ system with PRF, GZMB or PRF+GZMB ART cells. **(g)** Summary of four different cytotoxicity experiments. **(h)** Skipping of exon 2 of PRF mRNA in T cells obtained from chronic hepatitis B patients and transfected with PRF SSO. **(i)** Flow cytometry staining of PRF in PRF ART cells from CHB patients. *p < 0.05, **p < 0.01, ***p < 0.001. GZMB, granzyme B; PRF, perforin. Color images are available online.

To further test the ability of SSOs to modulate essential T cell functions, we designed SSOs to suppress the expressions of *PRF1* and *GZMB*. For the former, the PRF SSO induces the skipping of exon 2b where the translation start codon resides, whereas the GZMB SSO induces exon 3 skipping that generates a frameshifted transcript (Fig. 3a); in both cases, no protein product is expected from the respective resultant transcripts. We quantified the exon skipping efficiency of each SSO 24 h after electroporation as PSI, shown in Fig. 3b and d. Intracellular cytokine staining (ICS) of PRF and GZMB ART cells shows a reduction of the respective proteins (Fig. 3c, e and Supplementary Fig. S3e). Again, both PRF and GZMB SSOs do not affect T cell viability (Supplementary Fig. S3a, b) and TCR expression (Supplementary Fig. S3c, d) compared with the controls, but inhibit their cytolytic ability (Fig. 3f, g) in T cells cotransfected with both the SSOs. Of importance, modulation of T cell function with SSOs was not demonstrated only on T cells of healthy individuals but on T cells of patients with inherent pathology (ie, chronic hepatitis B infection) (Fig. 3h, i, and Supplementary Fig. S3f).

## Discussion

To engineer ART cells with definite function and specificity, we decided to utilize fully chemically modified splicing-modulating SSOs over siRNA, GAPmer, shRNA, and CRISPR/Cas9 approaches, for the following reasons: (i) superior selectivity and stability, (ii) multimodality, (iii) limited immunogenicity, and (iv) clinical compatibility owing to their transient nature and their success in clinic [36 37,^51^,^52^]. Thanks to their versatility, SSOs could be explored in other cell types also used in adoptive transfer (such as NK cells or dendritic cells) to further ameliorate cell-based therapeutic approaches. The manipulation *ex vivo* of cells used for adoptive cell transfer is relatively straightforward. In fact, high numbers of PBMCs can be easily obtained through phlebotomy and they can be expanded *in vitro* for several days before reinfusion into the patient. During the expansion phase, concurrent modifications can be implemented for intrinsic engineering of these cells: from redirection, to the boosting of their function using cytokines or other drugs [53,54].

The advantage of our cotransfection protocol is evident when considering its practicality in a clinical setting, where substantial and subsequent manipulations of the samples could lead to higher risk of contamination and/or loss of the sample itself. The choice of transfection (over transduction) offers an advantage with regard to safety. Transient redirection is safer for the patient, as the specificity receptor is lost within few days, with lower risk of collateral effects [8]. After the exogenous specificity receptor is lost, T cells still maintain their natural receptor; therefore, any stable functional modification induced would alter indefinitely their function against the natural targets. Another advantage of an SSO lies in its noncatalytic action, which does not require a functional RNAi machinery or RNase H activity, unlike siRNA and GAPmer, respectively [40]. Given the patient variability, potential deregulation of the two endogenous cellular factors in exhausted primary T cells and cytotoxicity effects from dose-induced saturation of these factors [55,56], anticipating the efficacy and the toxicity of both siRNA and GAPmer is not straightforward.

Our data showed that SSO-mediated intrinsic engineering of primary human T cells from healthy donors and chronic hepatitis B patients does not impinge on the ART cell viability and TCR expression, as well as T cell antiviral or cytotoxic function mediated by the extrinsic engineering. On the contrary, we were able to modify immune-modulatory and cytotoxic functions of ART cells targeting IFN-γ, PRF, and GZMB production singly and in combination, and in conjunction with extrinsic engineering.

In summary, this work is a technical demonstration that targeted intrinsic engineering of immune cells for adoptive immunotherapy is possible with splice-switching oligonucleotides. This work paves the way for more applications in the field, as well as, a clinical translation of the technology.

## Supplementary Material

Supplemental data

Supplemental data

Supplemental data

## References

[1] Anthony-gonda K, A Bardhi, A Ray, N Flerin, M Li, W Chen, C Ochsenbauer, JC Kappes, W Krueger, *et al.* (2019). Multi-specific anti-HIV duoCAR-T cells display broad antiviral activity and potent elimination of HIV-infected cells in vivo. Sci Transl Med 11: eaav5685 10.1126/scitranslmed.aav5685 PMC7136029 31391322

[2] Krebs K, N Böttinger, LR Huang, M Chmielewski, S Arzberger, G Gasteiger, C Jäger, E Schmitt, F Bohne, *et al.* (2013). T cells expressing a chimeric antigen receptor that binds hepatitis B virus envelope proteins control virus replication in mice. Gastroenterology 145:456–465 23639914 10.1053/j.gastro.2013.04.047

[3] Sautto GA, K Wisskirchen, N Clementi, M Castelli, RA Diotti, J Graf, M Clementi, R Burioni, U Protzer, and N Mancini. (2016). Chimeric antigen receptor (CAR)-engineered T cells redirected against hepatitis C virus (HCV) E2 glycoprotein. Gut 65:512–523 25661083 10.1136/gutjnl-2014-308316 PMC4789830

[4] Kah J, S Koh, T Volz, E Ceccarello, L Allweiss, M Lütgehetmann, A Bertoletti, and M Dandri. (2017). Lymphocytes transiently expressing virus-specific T cell receptors reduce hepatitis B virus infection. J Clin Invest 127:3177–3188 28737510 10.1172/JCI93024 PMC5531408

[5] Proff J, CU Brey, A Ensser, W Holter, and M Lehner. (2018) Turning the tables on cytomegalovirus: targeting viral Fc receptors by CARs containing mutated CH2-CH3 IgG spacer domains. J Transl Med 16:1–12 29422056 10.1186/s12967-018-1394-x PMC5804023

[6] Kumaresan PR, PR Manuri, ND Albert, S Maiti, H Singh, T Mi, J Roszik, B Rabinovich, S Olivares, *et al.* (2014). Bioengineering T cells to target carbohydrate to treat opportunistic fungal infection. Proc Natl Acad Sci USA 111:10660–10665 25002471 10.1073/pnas.1312789111 PMC4115509

[7] Varela-Rohena A, PE Molloy, SM Dunn, Y Li, MM Suhoski, RG Carroll, A Milicic, T Mahon, DH Sutton, *et al.* (2008). Control of HIV-1 immune escape by CD8 T cells expressing enhanced T-cell receptor. Nat Med 14:1390–1395 18997777 10.1038/nm.1779 PMC3008216

[8] Bertoletti A and AT Tan. (2020). Challenges of CAR- And TCR-T cell-based therapy for chronic infections. J Exp Med 217:4–6 10.1084/jem.20191663 PMC7201928 32163104

[9] Friedman KM, TE Garrett, JW Evans, HM Horton, HJ Latimer, SL Seidel, CJ Horvath, and RA Morgan. (2018). Effective targeting of multiple B-cell maturation antigen-expressing hematological malignances by Anti-B-cell maturation antigen chimeric antigen receptor T cells. Hum Gene Ther 29:585–601 29641319 10.1089/hum.2018.001 PMC5930946

[10] Maude SL, TW Laetsch, J Buechner, S Rives, M Boyer, H Bittencourt, P Bader, MR Verneris, HE Stefanski, *et al.* (2018). Tisagenlecleucel in Children and Young Adults with B-Cell Lymphoblastic Leukemia. N Engl J Med 378:439–448 29385370 10.1056/NEJMoa1709866 PMC5996391

[11] Tan AT, N Yang, TL Krishnamoorthy, V Oei, A Chua, Z Xinyuan, TH Si, A Chia, N Le Bert, *et al.* (2019). Use of expression profiles of HBV-DNA integrated into genomes of hepatocellular carcinoma cells to select T cells for immunotherapy. Gastroenterology 156:1–15 10.1053/j.gastro.2019.01.251 30711630

[12] Draper LM, MLM Kwong, A Gros, S Stevanović, E Tran, S Kerkar, M Raffeld, SA Rosenberg, and CS Hinrichs. (2015). Targeting of HPV-16+epithelial cancer cells by TCR gene engineered T cells directed against E6. Clin Cancer Res 21:4431–4439 26429982 10.1158/1078-0432.CCR-14-3341 PMC4603283

[13] Kato D, T Yaguchi, T Iwata, Y Katoh, K Morii, K Tsubota, Y Takise, M Tamiya, H Kamada *et al.* (2020). GPC1 specific CAR-T cells eradicate established solid tumor without adverse effects and synergize with anti-PD-1 ab. Elife 9:1–20 10.7554/eLife.49392 PMC7108862 32228854

[14] Brown CE, D Alizadeh, R Starr, L Weng, JR Wagner, A Naranjo, JR Ostberg, MS Blanchard, J Kilpatrick, *et al.* (2016). Regression of glioblastoma after chimeric antigen receptor T-cell therapy. N Engl J Med 375:2561–2569 28029927 10.1056/NEJMoa1610497 PMC5390684

[15] Schuberth PC, C Hagedorn, SM Jensen, P Gulati, M van den Broek, A Mischo, A Soltermann, A Jüngel, O Marroquin Belaunzaran, *et al.* (2013). Treatment of malignant pleural mesothelioma by fibroblast activation protein-specific re-directed T cells. J Transl Med 11:1–11 23937772 10.1186/1479-5876-11-187 PMC3751305

[16] Majzne RG, JL Theruvath, A Nellan, S Heitzeneder, Y Cui, CW Mount, SP Rietberg, MH Linde, P Xu, *et al.* (2019). CAR T cells targeting B7-H3, a pan-cancer antigen, demonstrate potent preclinical activity against pediatric solid tumors and brain tumors. Clin Cancer Res 25:2560–2574 30655315 10.1158/1078-0432.CCR-18-0432 PMC8456711

[17] Morgan RA, N Chinnasamy, D Abate-Daga, A Gros, PF Robbins, Z Zheng, ME Dudley, SA Feldman, JC Yang, *et al.* (2013). Cancer regression and neurological toxicity following anti-MAGE-A3 TCR gene therapy. J Immunother 36:133–151 23377668 10.1097/CJI.0b013e3182829903 PMC3581823

[18] Roth E and H Pircher. (2004). IFN- promotes Fas ligand- and perforin-mediated liver cell destruction by cytotoxic CD8 T Cells. J Immunol 172:1588–1594 14734739 10.4049/jimmunol.172.3.1588

[19] Rafiq S, OO Yeku, HJ Jackson, TJ Purdon, DG van Leeuwen, DJ Drakes, M Song, MM Miele, Z Li, *et al.* (2018). Targeted delivery of a PD-1-blocking scFv by CAR-T cells enhances anti-tumor efficacy in vivo. Nat Biotechnol 36:847–856 30102295 10.1038/nbt.4195 PMC6126939

[20] Hu B, J Ren, Y Luo, B Keith, RM Young, J Scholler, Y Zhao, and CH June. (2017). Augmentation of antitumor immunity by human and mouse CAR T cells secreting IL-18. Cell Rep 20:3025–3033 28954221 10.1016/j.celrep.2017.09.002 PMC6002762

[21] Pegram HJ, JC Lee, EG Hayman, GH Imperato, TF Tedder, M Sadelain, and RJ Brentjens. (2012).“Tumor-targeted T cells modified to secrete IL-12 eradicate systemic tumors without need for prior conditioning. Blood 119:4133–4141 22354001 10.1182/blood-2011-12-400044 PMC3359735

[22] Ligtenberg MA, Y Pico de Coaña, T Shmushkovich, Y Yoshimoto, I Truxova, Y Yang, M Betancur-Boissel, AV Eliseev, AD Wolfson, and R Kiessling. (2018). Self-Delivering RNAi Targeting PD-1 improves tumor-specific T cell functionality for adoptive cell therapy of malignant melanoma. Mol Ther 26:1482–1493 29735366 10.1016/j.ymthe.2018.04.015 PMC5986970

[23] Newick K, S O'Brien, J Sun, V Kapoor, S Maceyko, A Lo, E Puré, E Moon, and SM Albelda. (2016). Augmentation of CAR T-cell trafficking and antitumor efficacy by blocking protein kinase a localization. Cancer Immunol Res 4:541–551 27045023 10.1158/2326-6066.CIR-15-0263 PMC4891259

[24] Qasim W, H Zhan, S Samarasinghe, S Adams, P Amrolia, S Stafford, K Butler, C Rivat, G Wright, *et al.* (2017). Molecular remission of infant B-ALL after infusion of universal TALEN gene-edited CAR T cells. Sci Transl Med 9:eaaj2013 28123068 10.1126/scitranslmed.aaj2013

[25] Stadtmauer EA, JA Fraietta, MM Davis, AD Cohen, KL Weber, E Lancaster, PA Mangan, I Kulikovskaya, M Gupta, *et al.* (2020). CRISPR-engineered T cells in patients with refractory cancer. Science 367:1–20 10.1126/science.aba7365 PMC11249135 32029687

[26] Zou F, L Lu, J Liu, B Xia, W Zhang, Q Hu, W Liu, Y Zhang, Y Lin, *et al.* (2019). Engineered triple inhibitory receptor resistance improves anti-tumor CAR-T cell performance via CD56. Nat Commun 10:1–14 31511513 10.1038/s41467-019-11893-4 PMC6739330

[27] Zhao L, Y Liu, F Zhao, Y Jin, J Feng, R Geng, J Sun, L Kang, L Yu, and Y Wei. (2020). Inhibition of cholesterol esterification enzyme enhances the potency of human chimeric antigen receptor T cells against pancreatic carcinoma. Mol Ther Oncolytics 16:262–271 32181327 10.1016/j.omto.2020.01.008 PMC7063140

[28] Watanabe N, T Nagata, Y Satou, S Masuda, T Saito, H Kitagawa, H Komaki, K Takagaki, and S Takeda. (2018). NS-065/NCNP-01: an antisense oligonucleotide for potential treatment of Exon 53 skipping in duchenne muscular dystrophy. Mol Ther Nucleic Acids 13:442–449 30388618 10.1016/j.omtn.2018.09.017 PMC6202794

[29] Rigo F, Y Hua, SJ Chun, TP Prakash, AR Krainer, and CF Bennett. (2016). Synthetic oligonucleotides recruit ILF2/3 to RNA transcripts to modulate splicing. Nat Chem Biol 8:555–561 10.1038/nchembio.939 PMC5021312 22504300

[30] Lin J, JHJ Lee, K Paramasivam, E Pathak, Z Wang, ZAD Pramono, B Lim, KB Wee, and U Surana. (2017). Induced-decay of glycine decarboxylase transcripts as an anticancer therapeutic strategy for non-small-cell lung carcinoma. Mol Ther Nucleic Acids 9:263–273 29246305 10.1016/j.omtn.2017.10.001 PMC5675722

[31] Georgilis A, S Klotz, CJ Hanley, N Herranz, B Weirich, B Morancho, AC Leote, L D'Artista, S Gallage, *et al.* (2018). PTBP1-mediated alternative splicing regulates the inflammatory secretome and the pro-tumorigenic effects of senescent cells. Cancer Cell 34:85–102 29990503 10.1016/j.ccell.2018.06.007 PMC6048363

[32] Koh CM, M Bezzi, DHP Low, WX Ang, SX Teo, FPH Gay, M Al-Haddawi, SY Tan, M Osato, *et al.* (2015). MYC regulates the core pre-mRNA splicing machinery as an essential step in lymphomagenesis. Nature 523:96–100 25970242 10.1038/nature14351

[33] Pao PW, KB Wee, WC Yee, and ZAD Pramono. (2014). Dual masking of specific negative splicing regulatory elements resulted in maximal exon 7 inclusion of SMN2 gene. Mol Ther 22:854–861 24317636 10.1038/mt.2013.276 PMC3982506

[34] Pramono ZAD, KB Wee, JL Wang, YJ Chen, QB Xiong, PS Lai, and WC Yee. (2012). A prospective study in the rational design of efficient antisense oligonucleotides for exon skipping in the DMD gene. Hum Gene Ther 23:781–790, 2012 10.1089/hum.2011.205 PMC3404420 22486275

[35] Wee KB, ZAD Pramono, JL Wang, KF MacDorman, PS Lai, and WC Yee. (2008). Dynamics of co-transcriptional pre-mRNA folding influences the induction of dystrophin exon skipping by antisense oligonucleotides. PLoS One 3:e1844 18365002 10.1371/journal.pone.0001844 PMC2267000

[36] Mercuri E, BT Darras, CA Chiriboga, JW Day, C Campbell, AM Connolly, ST Iannaccone, J Kirschner, NL Kuntz, *et al.* (2018). Nusinersen versus sham control in later-onset spinal muscular atrophy. N Engl J Med 378:625–635 29443664 10.1056/NEJMoa1710504

[37] Kim J, C Hu, C Moufawad El Achkar, LE Black, J Douville, A Larson, MK Pendergast, SF Goldkind, EA Lee, *et al.* (2019). Patient-customized oligonucleotide therapy for a rare genetic disease. N Engl J Med 381:1644–1652, 2019 10.1056/NEJMoa1813279 PMC6961983 31597037

[38] Zhou Y, C Han, E Wang, AH Lorch, V Serafin, BK Cho, BT Guttierrez Diaz, J Calvo, C Fang, *et al.* (2020). Posttranslational regulation of the exon skipping machinery controls aberrant splicing in leukemia. Cancer Discov 10:1388–1409 32444465 10.1158/2159-8290.CD-19-1436 PMC7483384

[39] Tabaglio T, DHP Low, WKL Teo, PA Goy, P Cywoniuk, H Wollmann, J Ho, D Tan, J Aw, *et al.* (2018). MBNL1 alternative splicing isoforms play opposing roles in cancer. Life Sci Alliance 1:1–14 10.26508/lsa.201800157 PMC6238595 30456384

[40] Do DV, B Strauss, E Cukuroglu, I Macaulay, KB Wee, TX Hu, I Ruiz De Los Mozos, C Lee, A Harrison, *et al.* (2018). SRSF3 maintains transcriptome integrity in oocytes by regulation of alternative splicing and transposable elements. Cell Discov 4:33 29928511 10.1038/s41421-018-0032-3 PMC6006335

[41] Toh CXD, JW Chan, ZS Chong, HF Wang, HC Guo, S Satapathy, D Ma, GYL Goh, E Khattar, *et al.* (2016). RNAi reveals phase-specific global regulators of human somatic cell reprogramming. Cell Rep 15:2597–2607 27292646 10.1016/j.celrep.2016.05.049

[42] Koh S, N Shimasaki, R Suwanarusk, ZZ Ho, A Chia, N Banu, SW Howland, ASM Ong, AJ Gehring, *et al.* (2013). A practical approach to immunotherapy of hepatocellular carcinoma using T cells redirected against hepatitis B virus. Mol Ther Nucleic Acids 2:e114 23941866 10.1038/mtna.2013.43 PMC3759740

[43] Koh S, J Kah, CYL Tham, N Yang, E Ceccarello, A Chia, M Chen, A Khakpoor, A Pavesi, *et al.* (2018). Nonlytic lymphocytes engineered to express virus-specific T-cell receptors limit HBV infection by activating APOBEC3. Gastroenterology 155:180–193 29550589 10.1053/j.gastro.2018.03.027

[44] Mann CJ, XM Anguela, J Montané, M Obach, C Roca, A Ruzo, P Otaegui, LM Mir, and F Bosch. (2012). Molecular signature of the immune and tissue response to non-coding plasmid DNA in skeletal muscle after electrotransfer. Gene Ther 19:1177–1186 22170344 10.1038/gt.2011.198

[45] Schroder K, P Hertzog, T Ravasi, and DA Hume. (2004). Interferon gamma: an overview of signals, mechanisms and functions. J Leukoc Biol 75:163–189 14525967 10.1189/jlb.0603252

[46] Refaeli Y, L Van Parijs, SI Alexander, and AK Abbas. (2002). Interferon γ is required for activation-induced death of T lymphocytes. J Exp Med 196:999–1005 12370261 10.1084/jem.20020666 PMC2194022

[47] Garcia-Diaz A, DS Shin, B Homet Moreno, J Saco, H Escuin-Ordinas, G Abril-Rodriguez, JM Zaretsky, L Sun, W Hugo, *et al.* (2017). Interferon receptor signaling pathways regulating PD-L1 and PD-L2 expression. Cell Rep 19:1189–1201 28494868 10.1016/j.celrep.2017.04.031 PMC6420824

[48] Kruger S, M Ilmer, S Kobold, BL Cadilha, S Endres, S Ormanns, G Schuebbe, BW Renz, JG D'Haese, *et al.* (2019). Advances in cancer immunotherapy 2019- latest trends. J Exp Clin Cancer Res 38:1–11 31217020 10.1186/s13046-019-1266-0 PMC6585101

[49] Lee SWL, G Adriani, E Ceccarello, A Pavesi, AT Tan, A Bertoletti, RD Kamm, and SC Wong. (2018). Characterizing the role of monocytes in T cell cancer immunotherapy using a 3D microfluidic model. Front Immunol 9:416 29559973 10.3389/fimmu.2018.00416 PMC5845585

[50] Ni L and J Lu. (2018). Interferon gamma in cancer immunotherapy. Cancer Med 7:4509–4516 30039553 10.1002/cam4.1700 PMC6143921

[51] Aartsma-Rus A, AAM Janson, WE Kaman, M Bremmer-Bout, GJB Van Ommen, JT den Dunnen, and JCT van Deutekom. (2004). Antisense-induced multiexon skipping for duchenne muscular dystrophy makes more sense. Am J Hum Genet 74:83–92 14681829 10.1086/381039 PMC1181915

[52] Bauman J, N Jearawiriyapaisarn, and R Kole. (2009). Therapeutic potential of splice-switching oligonucleotides. Oligonucleotides 19:1–13 19125639 10.1089/oli.2008.0161 PMC2663420

[53] Barrero MJ. (2017). Epigenetic strategies to boost cancer immunotherapies. Int J Mol Sci 18:1108 10.3390/ijms18061108 PMC5485932 28545238

[54] Roberti MP, MM Barrio, AI Bravo, YS Rocca, JM Arriaga, M Bianchini, J Mordoh, and EM Levy. (2011). IL-15 and IL-2 increase Cetuximab-mediated cellular cytotoxicity against triple negative breast cancer cell lines expressing EGFR. Breast Cancer Res Treat 130:465–475 21308409 10.1007/s10549-011-1360-2

[55] Grimm D, L Wang, JS Lee, N Schürmann, S Gu, K Börner, TA Storm, and MA Kay. (2010). Argonaute proteins are key determinants of RNAi efficacy, toxicity, and persistence in the adult mouse liver. J Clin Invest 120:3106–3119 20697157 10.1172/JCI43565 PMC2929739

[56] Grimm D. (2011). The dose can make the poison: lessons learned from adverse *in vivo* toxicities caused by RNAi overexpression. Silence 2:8 22029761 10.1186/1758-907X-2-8 PMC3234190

